# BthTX-I from *Bothrops jararacussu* induces apoptosis in human breast cancer cell lines and decreases cancer stem cell subpopulation

**DOI:** 10.1590/1678-9199-JVATITD-2019-0010

**Published:** 2019-07-29

**Authors:** Patrícia Heloise Alves Bezerra, Isadora Marques Ferreira, Beatriz Tinoco Franceschi, Francine Bianchini, Luciana Ambrósio, Adélia Cristina O. Cintra, Suely Vilela Sampaio, Fabíola Attié de Castro, Maria Regina Torqueti

**Affiliations:** 1 Laboratory of Clinical Cytology, Department of Clinical Analyses, Toxicology and Food Science, School of Pharmaceutical Sciences of Ribeirão Preto, University of São Paulo (USP), Ribeirão Preto, SP, Brazil.; 2 Laboratory of Hematology, Department of Clinical Analyses, Toxicology and Food Science, School of Pharmaceutical Sciences of Ribeirão Preto, University of São Paulo (USP), Ribeirão Preto, SP, Brazil.; 3 Laboratory of Toxinology, Department of Clinical Analyses, Toxicology and Food Science, School of Pharmaceutical Sciences of Ribeirão Preto, University of São Paulo (USP), Ribeirão Preto, SP, Brazil.

**Keywords:** apoptosis, bothropstoxin, breast cancer, cancer stem cells

## Abstract

**Background:**

Breast cancer is the neoplasm with both the highest incidence and mortality rate among women worldwide. Given the known snake venom cytotoxicity towards several tumor types, we evaluated the effects of BthTX-I from *Bothrops jararacussu* on MCF7, SKBR3, and MDAMB231 breast cancer cell lines.

**Methods:**

BthTX-I cytotoxicity was determined via MTT 3-(4,5-dimethylthiazol-2-yl)-2,5-diphenyl-tetrazoliumbromide assay. Cell death was measured by a hypotonic fluorescent solution method, annexin-V-FITC/propidium iodide staining and by apoptotic/autophagic protein expression. Cancer stem cells (CSCs) were quantified by flow cytometry using anti-CD24-FITC and anti-CD44-APC antibodies and propidium iodide.

**Results:**

BthTX-I at 102 µg/mL induced cell death in all cell lines. The toxin induced apoptosis in MCF7, SKBR3, and MDAMB231 in a dose-dependent manner, as confirmed by the increasing number of hypodiploid nuclei. Expression of pro-caspase 3, pro-caspase 8 and Beclin-1 proteins were increased, while the level of the antiapoptotic protein Bcl-2 was diminished in MCF7 cells. BthTX-I changed the staining pattern of CSCs in MDAMB231 cells by increasing expression of CD24 receptors, which mediated cell death.

**Conclusions:**

BthTX-I induces apoptosis and autophagy in all breast cancer cell lines tested and also reduces CSCs subpopulation, which makes it a promising therapeutic alternative for breast cancer.

## Background

The incidence of cancer has increased over the last few decades, and it has become an evident global public health issue. The International Agency for Research on Cancer of the World Health Organization has predicted 18 million new neoplasm cases for 2018, among which breast cancer figures as the leading tumor type in women [1].

Breast cancer is a highly heterogeneous neoplasm and is classified according to the presence or absence of molecular biomarkers into luminal, HER-2-enriched, and triple-negative subtypes. The luminal subtype expresses hormonal receptors, i.e. estrogen and/or progesterone receptors; the HER-2-enriched subtype expresses neither estrogen nor progesterone receptors, however highly expresses the oncogene HER-2 (human epidermal growth factor receptor type 2); and the triple negative subtype does not express HER-2 or estrogen or progesterone receptors [2,3]. These breast cancer subtypes present different clinical outcomes: the luminal cancer responds well to therapy and has a good prognosis, while HER-2-enriched and triple-negative present a bad prognosis. 

Molecular subtyping of breast tumors is indispensable because it is directly associated with the therapeutic approach and the patient’s prognosis [2]. However, the ineffectiveness and high toxicity of chemotherapeutic drugs, and the fact that they are also associated with tumor resistance have limited breast cancer therapy and promoted a high demand for novel antitumor agents [4]. This has led to research using animal venoms and toxins, that have already demonstrated promising cytotoxic activity against many tumor types such as breast cancer, colorectal cancer, lung adenocarcinoma, melanoma, promyelocytic leukemia [5], chronic myeloid leukemia (CML) [6,7], and myeloproliferative neoplasm [8], both *in vitro* and *in vivo* [5]. 

Burin *et al.* [6,7] described the antileukemic effects of CR-LAAO and LAAO from *Bothops pirajai* (BpirLAAO-I) in BCR-ABL1-positive cells lines from CML patients. In addition, the toxin BpirLAAO-I was also able to activate immune cells and lymphocytes of healthy subjects, a process that is relevant for antitumor response in CML patients. Furthermore, BpirLAAO-I induced apoptosis and potentiated the tyronise kinase inhibitor effect on BCR-ABL^+^ cells. Additionally, Tavares *et al.* [8] reported an L-amino-acid oxidase from *C. rhodostoma* (CR-LAAO) snake venom as a potential antineoplastic agent against HEL 92.1.7 and SET-2 JAK2V617F cell lines derived from myeloproliferative neoplasm patients. Moreover, the cytotoxins CT1 and CT2 from *Naja oxiana*, CT3 from *Naja kaouthia* and CT1 from *Naja haje* showed an important cytotoxicity, mainly mediated by lysosome rupture, against lung adenocarcinoma A549 and promyelocytic leukemia HL60 cells [9]. 

In this context, the antitumor potential of bothropstoxin I (BthTX-I) was tested. BthTX-I is a phospholipase A_2_ (PLA_2_) from *Bothrops jararacussu* venom. BthTX-I, classified as a Lys-49-PLA_2_, is catalytically inactive and exerts myotoxic effects through mechanisms that are independent of binding to calcium channels [10,11]. BthTX-I has previously presented antitumor activity against HER-2^+^ breast cancer cells (SKBR3) [12,13]. Thus, the present study evaluated the antitumor potential of BthTX-I against MCF7, SKBR3, and MDAMB231 cell lines, which represent the luminal, HER-2-enriched, and triple-negative breast carcinoma subtypes, respectively. 

## Methods

### Cell culture

The MCF7 (luminal), SKBR3 (HER-2-enriched), and MDAMB231 (triple-negative) breast cancer cell lines were purchased from Rio de Janeiro Cell Bank (BCRJ, Rio de Janeiro, RJ, Brazil) and cultured in RPMI 1640 medium supplemented with 10% heat-inactivated FBS, 1% glutamine, 1% antibiotic/antimycotic solution, and incubated at 37 ºC under 5% CO_2_.

### Treatment of cell lines

The cell lines were treated with BthTX-I diluted in estrogen-free RPMI 1640 medium supplemented with charcoal stripped fetal bovine serum (CS-FBS) with increasing concentrations of the toxin (12, 25, 51, 102, 204, 409 µg/mL). As a positive control, cell lines were treated with one of three chemotherapeutic drugs (cisplatin at 100 µM, doxorubicin at 4 µM or N-desmethyltamoxifen at 20 µM). For the negative control, cells were treated only with estrogen-free RPMI 1640 medium supplemented with CS-FBS.

### Bothropstoxin-I (BthTX-I) purification

BthTX-I was purified from *B. jararacussu* venom by the Laboratory of Toxinology of the School of Pharmaceutical Sciences of Ribeirão Preto (USP).

*B. jararacussu* crude venom (150 mg) was fractionated by size-exclusion chromatography in a Shephacryl S-100 as described by Carone *et al.* [14]. The eluted fractions was monitored for absorbance at 280 nm, pooled, desalted in a Hi-prep 26/10 desalting column, and lyophilized. The fraction, denominated SPIII, was identified via SDS PAGE by containing phospholipases and myotoxins, which have molecular masses of approximately 14 to 17 kDa. For further purification, SPIII was submitted to ion exchange chromatography in a CM Sepharose column (40 x 2 cm; Amersham, GE Healthcare Life Science, USA), previously equilibrated with 50 mM ammonium bicarbonate pH 8 (Buffer A). Elution started with the same buffer, followed by a linear gradient of 500 mM ammonium bicarbonate pH 8 (Buffer B). The procedure was performed at a flow rate of 1.5 mL/min, with fractions of 4 mL collected based on absorbance at 280 nm. The fraction corresponding to BthTX-I was collected, lyophilized and stored at 4°C for subsequent analysis. The purity of BthTX-I was also assayed by 12% SDS-PAGE and by N-terminal amino-acid sequence, using automated Edman degradation in an automatic protein sequencer (PPSQ 33A, system, Shimadzu).

### Cell viability assay

The cellular viabilities of MCF7, SKBR3 and MDAMB231 were determined via MTT assay, as previously reported [15]. Cells were seeded into 96-well culture plates (2 × 10^4^ cells/well) and incubated overnight at 37°C under 5% CO_2_. The culture medium was removed and cells were treated with BthTX-I (12-409 μg/mL) or culture medium (negative control) for 24h. Twenty μL of a 5 mg/mL MTT solution was added to each well and plates were incubated for 4h at 37°C, under 5% CO_2_. The reaction medium was discarded; the formazan crystals were dissolved in DMSO, and the plates were incubated for 15 min. After shaking for 5 s, the absorbance was recorded at 570 nm in an Absorbance Microplate Reader Spectramax (Molecular Devices, USA). Cell viability results were expressed as percentages of the negative control. The IC_50_ values for each cell line were calculated using the software CompuSyn (CompuSyn Inc, USA), and at least three independent experiments were performed.

### Apoptosis and necrosis quantification

The rates of apoptosis and necrosis in MCF7, SKBR3, and MDAMB231 cell lines were quantified using the annexin V-FITC apoptosis detection kit and HFS assays. Annexin V-FITC detection was carried out according to the manufacturer’s instructions. Briefly, cells were seeded into 6-well culture plates (2-5 × 10^5^ cells/well), incubated overnight at 37°C, under 5% CO_2_, and further treated with BthTX-I (102 µg/mL) or culture medium (negative control) for 24h. Next, cells were washed with ice-cold PBS, suspended in 100 µL of binding buffer solution (25 mM CaCl_2_, 1.4 M NaCl, and 0.1 M Hepes/NaOH, pH 7.4), and incubated in the dark for 30 min with annexin V-FITC solution (1:100) and PI (1:150) at 100 µg/mL. The results of fluorescence-activated cell sorting (FACS) analysis, carried out in a FACS Canto II cytometer, were analyzed using the software FACSDiva (Becton Dickinson, USA). At least three independent tests were performed.

Apoptosis was also assessed using the hypotonic fluorescent solution (HFS) method [16]. Briefly, MCF7, SKBR3, and MDAMB231 cells were cultured in 6-well plates (2-5 × 10^5^/well) and treated with BthTX-I (10-200 μg/mL), chemotherapeutic drugs (positive control; 4 µM doxorubicin, 100 µM cisplatin, or 20 µM N-desmethyltamoxifen), or culture medium (negative control) for 12h. Cells were recovered, centrifuged at 240 × *g* for 5 min at 4°C, and suspended in 200 μL of HFS (1% Triton X-100, 1% sodium citrate, and 100 μg/mL of PI). After a 20-minute incubation period, cells were analyzed in the Canto II cytometer equipped with the software FACSDiva. The cell death percentage was calculated from the content of genomic DNA, meaning the percentage of hypodiploid nuclei. Five thousand events from the gate were acquired and analyzed by histogram. At least three independent tests were performed.

### Bcl-2, Beclin-1 and pro-caspase 3, 8 and 9 levels

Twenty-four hours after treatment with BthTX-I, MCF7 cells were harvested, washed, and lysed with lysis buffer (Tris HCl 20 mM pH 7.5, NaCl 150 mM, EDTA 1 mM, Igepal CA 630 1% v/v, sodium pyrophosphate 25 mM, sodium orthovanadate 10 mM, β-glycerophosphate 10 mM, protease-inhibitor cocktail). Protein concentration in the resulting lysate was determined using Pierce BCA Protein Quantification kit, as described by the manufacturer. Twenty-five µg of protein were resolved by electrophoresis in 12% Tris-glycine polyacrylamide gels and transferred to nitrocellulose membranes. The membranes were blocked with 5% skim milk in TBST (20 mM Tris-HCl pH 7.4, 150 mM NaCl, 0.05% Tween 20), and incubated overnight with the appropriate primary antibody at 1:1,000 or 1:5,000 dilution. The primary antibodies (anti-caspase-3 rabbit polyclonal antibody, anti-caspase-8 mouse monoclonal antibody, anti-human caspase-9 rabbit polyclonal antibody, anti-Bcl-2 rabbit monoclonal antibody and anti-Beclin-1 rabbit polyclonal antibody) were purchased from Cell Signaling Technology® (USA).

After washing, the membranes were incubated with the corresponding horseradish peroxidase-conjugated secondary antibody at 1:7,500 or 1:20,000 dilution in TBST. The secondary antibodies (peroxidase-conjugated AffiniPure anti-rabbit and peroxidase-conjugated AffiniPure anti-mouse) were acquired from Jackson ImmunoResearch (USA). The bound secondary antibody was detected using an Amersham ECL (enhanced chemiluminescence) plus detection reagent, associated with exposure to a light-sensitive film. The actin levels (determined in the same cell lysates) were employed as a protein loading control. The protein expression levels were quantified by densitometry using the software Image J (NIH, version 1.8.0_112) and normalized in relation to actin expression.

### CD44 and CD24 immunophenotyping

Cells were trypsinized, washed with PBS, pelleted by spinning down, and resuspended in PBS. Cells were then incubated with FITC-conjugated mouse anti-human CD44 monoclonal antibody and APC-conjugated mouse anti-human CD24 monoclonal antibody for 30 min, in an ice bath, in the dark, according to the manufacturer’s instructions (Becton Dickinson, USA). Next, a PI solution at 100 µg/mL was added to the cells, which were immediately analyzed in the Canto II cytometer equipped with the software FACSDiva. Positive and negative binding beads were used as the positive and negative controls, respectively. The cell subpopulations were calculated among living cells, and at least three independent tests were performed.

### Statistical analysis

Data were analyzed using the software GraphPad Prism 6.0^®^ (GraphPad Software, USA) and expressed as mean ± standard deviation (SD). Two groups were compared using the Student’s *t* test, while three or more groups were compared by one-way ANOVA combined with the Tukey *post-hoc* analysis. Statistical significance was indicated by p < 0.05.

## Results

### Isolation and purification of BthTX-I

The purification of BthTX-I was successfully carried out by the two chromatographic steps previously mentioned. After the first step, in Sephacryl S100 column, the fraction SPIII (Additional file 1A) was identified by means of SDS PAGE containing phospholipase A_2_ and myotoxins (Additional file 1A, insert). The SPIII fraction was concentrated and subsequently submitted to the CM-Sepharose column (Additional file 1B). BthTX-I was detected in the fifth fraction, producing a peak found to be highly pure when analyzed by SDS-PAGE (Additional file 1B, insert). BthTX-I was characterized by N-terminal amino-acid sequence obtained by automatic Edman degradation, presenting 20 amino-acid residues (SLFELGKMILQETGKNPAKS) that showed high identity (100%) in relation to the N-terminal sequence of the BthTX-I, as reported by Cintra *et al.* [17].

### BthTX-I decreases viability of breast cancer cells

**Figure 1.**
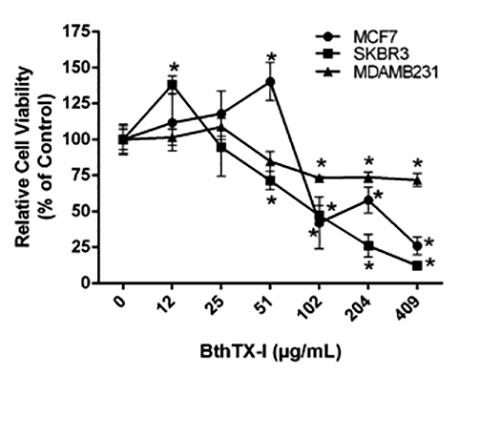
Viability of breast cancer cell lines treated with BthTX-I for 24h, assessed via MTT assay. Values represent the mean ± SD of at least three independent experiments. * p < 0.05 *vs.* negative control (ANOVA combined with the Tukey *post-hoc* test).

An MTT assay was used to examine the cytotoxicity of different BthTX-I concentrations towards MCF7, SKBR3, and MDAMB231 cells (Fig. 1). BthTX-I significantly decreased cell viability of MCF7, SKBR3, and MDAMB231 cells at concentrations greater than 102, 51, and 102 µg/mL, respectively, 24h after exposure. The toxin exerted a concentration-dependent cytotoxic effect only towards SKBR3 cells.

The BthTX-I concentration of 102 µg/mL was selected for subsequent trials because it was the minimum concentration that effectively reduced cell viability of all three cancer cell lines studied when compared with the negative control - it reduced cell viability of MCF7, SKBR3, and MDAMB231 cells by 57.97%, 52.82%, and 26.72%, respectively (p < 0.05). Moreover, SKBR3 cells were more sensitive to BthTX-I than MCF7 and MDAMB231 cells (Table 1).

**Table 1.** Cytotoxicity of BthTX-I towards breast cancer cell lines.

Cell line	IC_50_ (µg/mL)
MCF7	104.35 ± 13.21
SKBR3	81.20 ± 8.58
MDAMB231	> 409 ± 5.34

Cells were treated with BthTX-I for 24h and cell viability was determined via MTT assay. IC_50_ is the toxin concentration that inhibits cell growth by 50%. Values represent mean ± SD of at least three independent experiments.

### BthTX-I induces apoptosis and necrosis of breast cancer cells

**Figure 2.**
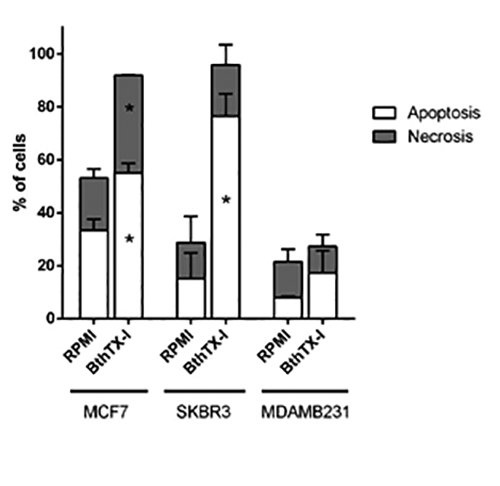
Cell death profile of breast cancer cell lines treated with BthTX-I at 102 µg/mL for 24h. Cell death was analyzed by the annexin V and propidium iodide staining flow cytometric method. RPMI: cells incubated in estrogen-free RPMI 1640 medium supplemented with CS-FBS (negative control). Values represent mean ± SD of at least three independent experiments. * p < 0.05 *vs.* negative control (Student’s *t* test).

Cell death profile of MCF7, SKBR3, and MDAMB231 cells treated with BthTX-I at 102 µg/mL for 24h was determined using the annexin-V and PI staining flow cytometric analysis (Fig. 2). Compared with the negative control (RPMI), BthTX-I significantly induced apoptosis of MCF7 and SKBR3 cells (increase of 65.13% and 404.45%, respectively; p < 0.05) and necrosis of MCF7 cells (increase of 86.80%; p < 0.05), but did not induce significant levels of apoptosis or necrosis in MDAMB231 cells.

### BthTX-I increases hypodiploid nuclei in breast cancer cells

**Figure 3.**
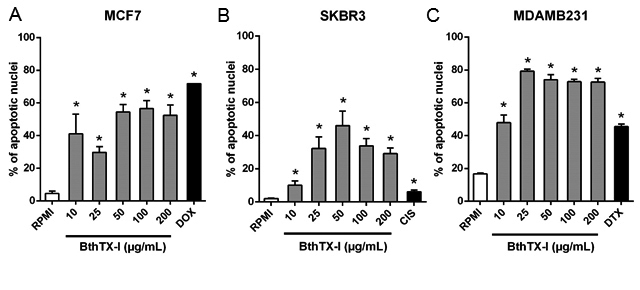
Percentages of hypodiploid nuclei in (**A**) MCF7, (**B**) SKBR3, and (**C**) MDAMB231 breast cancer cell lines treated with BthTX-I for 12h, through quantification of apoptotic nuclei by the hypotonic fluorescent solution method. Cells treated with (**A**) DOX (4 µM doxorubicin), (**B**) CIS (100 µM cisplatin), or (**C**) DTX (20 µM N-desmethyltamoxifen) were used as positive control. RPMI: cells incubated in estrogen-free RPMI 1640 medium supplemented with CS-FBS (negative control). Values represent mean ± SD of at least three independent experiments. * p < 0.05 *vs.* negative control (ANOVA combined with the Tukey *post-hoc* test). Ns: no significant difference between results in bracket (p > 0.05).

To address whether apoptosis induction was the main mechanism of cell death in the breast cancer cell lines in response to BthTX-I, hypodiploid nuclei (apoptotic cells) were quantified using the HFS staining flow cytometric assay (Fig. 3). Twelve hours after BthTX-I treatment at concentrations as low as 10 µg/mL, a significantly greater formation of hypodiploid nuclei in MCF7, SKBR3 and MDAMB231 cells was observed when compared with the negative control (increase of 38.20%, 8.27%, and 31.13% respectively; p < 0.05). The toxin induced apoptosis of the three cell lines in a dose-dependent manner and reached a peak at respective concentrations of 50, 100 and 25 µg/mL in MCF7, SKBR3 and MDAMB231 cells. Therefore, apoptosis induction was the main mechanism by which BthTX-I caused death of breast cancer cells.

### BthTX-I strongly induces expression of proapoptotic and proautophagic proteins in MCF7 cells

**Figure 4.**
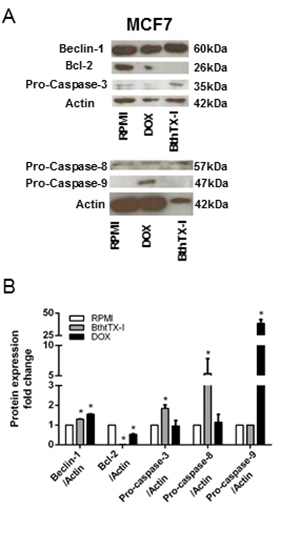
Beclin-1 and Bcl-2 protein (**A**) expression and (**B**) quantification in MCF7 cells treated with BthTX-I at 102 µg/mL for 24h. DOX: cells treated with doxorubicin at 4 µM (positive control). RPMI: cells incubated in estrogen-free RPMI 1640 medium supplemented with CS-FBS (negative control). * p < 0.05 *vs* negative control (Student’s *t* test).

The expression levels of proapoptotic (pro-caspase 3, 8 and 9), antiapoptotic (Bcl-2), and proautophagic (Beclin-1) proteins in MCF7 cells treated with BthTX-I at 102 µg/mL for 24h were quantified by Western Blot analysis (Fig. 4). Compared with the negative control, BthTX-I upregulated the expression of pro-caspase 3, pro-caspase 8 and of Beclin-1 (increase of 83.94%, 535%, and 29.57%, respectively). In addition, the toxin also downregulated the expression of Bcl-2 (reduction of 99.97%). BthTX-I did not alter the levels of pro-caspase 9 expression. Doxorubicin was used as positive control for apoptosis and autophagy induction.

### CD44/CD24 expression levels on breast cancer cell lines treated with BthTX-I

**Figure 5.**
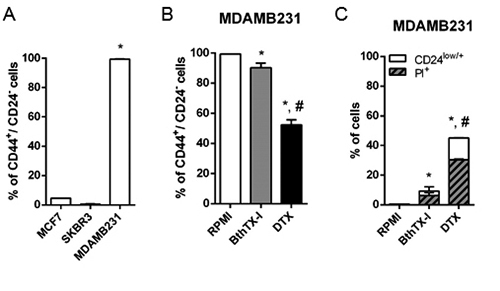
Quantification of cancer stem cells. The CSCs immunophenotyping quantification was carried out by flow cytometry using propidium iodide staining of the biomarkers CD24 and CD44. (**A**) Percentage of CD44^+^/CD24^-^ cancer stem cells in untreated breast cancer cell lines. (**B**) Percentage of CD44^+^/CD24^-/low^ cancer stem cells in the MDAMB231 cell population treated with BthTX-I at 102 µg/mL for 24h. (**C**) Expression of CD24^+^ cells in the cell population depicted in (**B**). DTX: cells treated with 20 µM N-desmethyltamoxifen (positive control). RPMI: cells incubated in estrogen-free RPMI 1640 medium supplemented with CS-FBS (negative control). PI: Propidium iodide staining. Values represent mean ± SD of at least three independent experiments. p < 0.05 *vs.* *negative control and *vs.* ^#^BthTX-I (Student’s *t* test).

To identify the cancer stem cell (CSC) subpopulation, we analyzed expression of the markers CD44 and CD24 by flow cytometry (Fig. 5). First, the breast carcinoma cell lines were characterized as CD44^+^/CD24^-/low^. Second, analysis of this subpopulation among the three cell lines revealed that the MDAMB231 cell line presented the greatest CSC prevalence (99.3%) when compared with the MCF7 and SKBR3 cell lines (4.5% and 0.55%, respectively) (Fig. 5A). Compared with the negative control, treatment with BthTX-I changed the expression pattern of a significant number of MDAMB231 cells (9.08% cells; p < 0.05) from CD44^+^/CD24^-^ to CD44^+^/CD24^low^ (Fig. 5B). Although CD44^+^/CD24^low^ cells are still classified as CSCs, most of them became PI^+^ (6.23%) (Fig. 5C), indicating that this alteration in CD24 expression resulted in cell death. We used binding beads and N-desmethyltamoxifen as the CD24 binding control and positive control, respectively (Additional file 2).

## Discussion

Previous studies have demonstrated the cytotoxicity of snake venoms towards several tumor types [5,18]. This property is attributed to the phospholipase A_2_ (PLA_2_) class of enzymes, independently of their catalytic capability. Some PLA_2_s from snakes belonging to the genus *Bothrops* exert antineoplastic action, such as myotoxin-II from *Bothrops asper* that is cytotoxic to adrenal tumor, and MjTX-II from *Bothrops moojeni* venom that is cytotoxic to Ehrlich ascites tumor, SKBR3 breast adenocarcinoma, and Jurkat T-cell leukemia [19]. Furthermore, a prior study found that MjTX-I, a PLA_2_ from *Bothrops moojeni*, is able to diminish cell viability of chronic myeloid leukemia cells (K562-S and K562-R BCR-ABL^+^) and induce apoptosis through activation of Caspases 3, 8 and 9 [20]. It remains unclear exactly how bothropstoxins from *B. jararacussu* affect mammary carcinomas.

Gebrim *et al.* [13] have shown that BthTX-I is cytotoxic to 75-90% of B16F10 melanoma cells, Jurkat T-cell leukemia cells, and *in vivo* solid tumor (S180), but to only 45% of SKBR3 breast cancer cells [13]. Moreover, the same study found that a BthTX-I modified with *p*-bromophenacyl bromide (BPB-BthTX-I) and with a peptide synthesized from the C-terminal part of this toxin (pep-BthTX-I), exhibited an increased cytotoxicity to cancer cells and reduced myotoxicity [13]. Our findings show that BthTX-I at 102 µg/mL strongly decreased cell viability of SKBR3 cells (52.82%), which corroborates the finding of Gebrim *et al.* [13]. Herein, we have also reported the cytotoxicity of BthTX-I towards luminal (MCF7) and triple-negative (MDAMB231) breast cancer cell line subtypes (with respective decreases in cell viability of 57.98% and 26.72%). 

It is well known that apoptosis happens when cell death is genetically programed and not accidental [21,22]. In general, it is mediated by the action of genes and proteins that activate effector caspases (cysteine-dependent aspartate-specific proteases), and occurs through pathways that do not elicit an inflammatory response. As apoptosis is an innate antitumor mechanism, it is a limiting factor in neoplastic diseases so that its induction is a target of many chemotherapeutic drugs [21,22].

The present study demonstrated that the antineoplastic activity of BthTX-I is mainly mediated by apoptosis induction. The BthTX-I in MCF7 cells promoted phosphatidylserine externalization and hypodiploid nuclei formation, as well as augmented expression of two pro-apoptotic proteins (pro-caspases 3 and 8) and diminished expression of the anti-apoptotic protein Bcl-2. These findings corroborate previous reports that BthTX-I induces apoptosis in HL-60, human promyelocytic leukemia cell line and liver carcinoma cells (HepG2) [23]. Furthermore, other PLA_2_ toxins from snakes of the genus *Bothrops* may trigger apoptosis of tumor cells, such as BnSP-6 from *Bothrops pauloensis*, which elicits phosphatidylserine externalization, upregulates expression of the pro-apoptotic caspase 8 gene and downregulates expression of the anti-apoptotic Bcl-2, Bcl2l1, and BIRC-5 genes in MDAMB231 triple-negative breast cancer cells [24]. Additionally, in contrast to a report in the literature that MCF7 cells do not express caspase 3 [25], our findings suggest that BthTX-I can restore pro-caspase 3 expression and re-sensitize MCF7 cells to apoptosis, since this type of restoration was previously observed after exposure to another natural compound, curcumin [26]. 

Autophagy differs from apoptosis with respect to cell morphology and is characterized by degradation of cellular components and formation of vacuoles [21]. The initial autophagy stage is mediated by the emergence of membranes involved in the targeting of cytoplasm portions and organelles to form autophagosomes, which fuse with lysosomes; lysosomal enzymes degrade the autophagosome contents. It is well described in the literature that autophagy depends on the proteins Atg6 (Beclin-1), Atg5 and Atg7 [27]. As BthTX-I increased Beclin-1 expression, we concluded that this toxin induced autophagy conjointly with apoptosis in the breast cancer cell lines herein investigated. These results are in accord with literature reports that some PLA_2_s, such as BnSP-6 from *Bothrops pauloensis*, stimulate autophagy pathways that induce the formation of autophagic vacuoles in triple-negative breast cancer cells (MDAMB231) [24], and that crotoxin stimulates the formation of autophagic vacuoles in luminal breast cancer cells (MCF7) [28].

In breast tumors, the cancer stem cells (CSC) can be identified by the expression of CD44 and by low or absent expression of CD24 (CD44^+^/CD24^-/low^) [29,30]. CSCs are usually associated with resistance to chemotherapy and neoplasm relapse [31]. We found that most of the triple-negative breast cancer cells (MDAMB231) behaved as CSCs, while luminal (MCF7) and HER-2-enriched (SKBR3) cell populations did not show an expressive CSC subpopulation. This result corroborates the findings reported by Ricardo *et al.* [32] and reinforces the high aggressiveness of triple-negative breast cancer in the clinic. Additionally, our IC_50_ results showed that BthTX-I is more efficient in diminishing cell viability in HER-2-enriched breast cancer subtype, followed by luminal and then by triple-negative. Thus, we speculate that the lessened effect of BthTX-I on triple-negative subtype is attributable to the higher aggressiveness of this tumor.

We also found that treatment of MDAMB231 cells with BthTX-I altered the CSC staining pattern by increasing CD24 expression, which in turn reduced cell viability. The literature reports that CD24 expression is lower in progenitor cells than in differentiated cells. Although some authors state that the presence of CD24 promotes cell proliferation and survival, other authors conclude that the presence of CD24 suppresses invasion and metastasis [33].

The findings reported herein also suggest that BthTX-I promotes autophagy by upregulating expression of Beclin-1 in MCF7 cells. It has already been demonstrated that C2 ceramide, a known autophagy inductor, decreases the CD44^+^/CD24^-/low^ cell subpopulation in breast cancer cells (MCF7) and larynx cancer cells (Hep-2), indicating that activation of this cell-death pathway may be a mechanism to diminish the cancer-cell subpopulation that is resistant to conventional therapy [34].

Taken together, ours results suggest that BthTX-I exerts a noticeable cytotoxicity towards luminal (MCF7), HER-2-enriched (SKBR3), and triple-negative (MDAMB231) breast-cancer-cell lines. This toxin not only induces cell death mainly via autophagy and the apoptosis pathway, but also decreases the number of MDAMB231 CSC - which are associated with resistance to chemotherapeutic drugs, cancer relapse, and aggressiveness of triple-negative neoplasms. Therefore, BthTX-I is a promising candidate for therapy against breast tumors that deserves to be tested *in vivo*.

### Abbreviations

HER-2: human epidermal growth factor receptor-type 2; BthTX-I: bothropstoxin I; PLA_2_: phospholipase A_2_; RPMI-1640: Roswell Park Memorial Institute 1640; FBS: fetal bovine serum; CML: chronic myeloid leucemia; CS-FBS: charcoal-stripped fetal bovine serum; MTT: 3-(4,5-dimethylthiazol-2-yl)-2,5-diphenyl-tetrazoliumbromide; DMSO: dimethyl sulfoxide; PI: propidium iodide; FACS: fluorescence-activated cell sorting; HFS: hypotonic fluorescent solution; SD: standard deviation; IC_50_: half maximal inhibitory concentration; CSC: cancer stem cells; Caspase: cystein-dependent aspartate-specific protease.

## Supplementary material

The following online material is available for this article:

Additional file 1. Isolation of BthTX-I from *Bothrops jararacussu* venom. (A) Chromatographic profile of *B. jararacussu* crude venom (150 mg) on Sephacryl S 100 column under elution with 20 mM Tris Hcl + 150 mM NaCl, pH 8. Fraction of 1 mL was collected at a flow rate of 12 mL/h, at room temperature. Inset: 12% SDS- PAGE of SPIII fraction under reducing conditions (1); molecular mass standards (2). Purification of BthTX-I. (B) Chromatography of 20 mg of SPIII fraction on CM-Sepharose previously equilibrated with 50 mM ammonium bicarbonate, pH 8, and then eluted on a concentration gradient of up to 50 mM of the same buffer. Fraction of 4 mL was collected at a flow rate of 1.52 mL/min, at room temperature. Inset: 12% SDS- PAGE of BthTX-I under reducing conditions (2); molecular mass standards (1).

Additional file 1. Flow cytometric analysis of cancer stem cell subpopulation in MDAMB231 cells treated with BthTX-I at 102 µg/mL for 24h. The CD24 and CD44 markers were quantified and antibody testing was performed with Beads (control). DTX: N-desmethyltamoxifen at 20 µM (positive control). RPMI: cells incubated in estrogen-free RPMI 1640 medium supplemented with CS-FBS (negative control).
