# Supplementary material for: BthTX-I from Bothrops jararacussu induces apoptosis in human breast cancer cell lines and decreases cancer stem cell subpopulation
Source: J Venom Anim Toxins Incl Trop Dis. 2019 Jul 29;25:e20190010. doi: 10.1590/1678-9199-JVATITD-2019-0010 (PMC6665320; doi:10.1590/1678-9199-JVATITD-2019-0010)
Supplement: Additional file 1. [file 1678-9199-jvatitd-25-e20190010-s1.pdf]

Supplementary Material to "BthTX-I from *Bothrops jararacussu* induces apoptosis in human breast cancer cell lines and decreases cancer stem cell subpopulation"

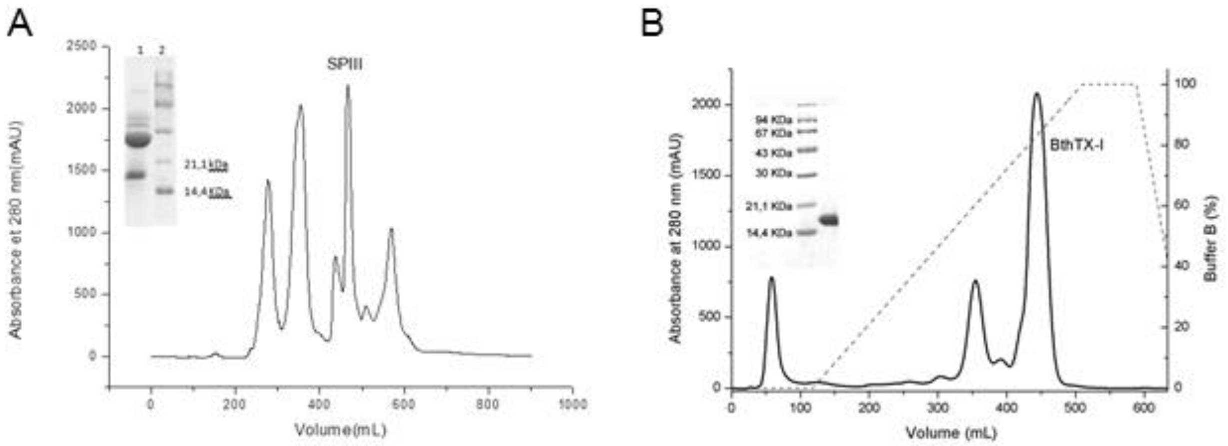

Additional file 1. Isolation of BthTX-I from *Bothrops jararacussu* venom. (**A**) Chromatographic profile of *B. jararacussu* crude venom (150 mg) on Sephacryl S 100 column under elution with 20 mM Tris Hcl + 150 mM NaCl, pH 8. Fraction of 1mL was collected at a flow rate of 12 mL/h, at room temperature. Inset: 12% SDS- PAGE of SPIII fraction under reducing conditions (1); molecular mass standards (2). Purification of BthTX-I. **(B)** Chromatography of 20 mg of SPIII fraction on CM-Sepharose previously equilibrated with 50 mM ammonium bicarbonate, pH 8, and then eluted on a concentration gradient of up to 50 mM of the same buffer. Fraction of 4 mL was collected at a flow rate of 1.52 mL/min, at room temperature. Inset: 12% SDS- PAGE of BthTX-I under reducing conditions (2); molecular mass standards (1).
