# Supplementary material for: BthTX-I from Bothrops jararacussu induces apoptosis in human breast cancer cell lines and decreases cancer stem cell subpopulation
Source: J Venom Anim Toxins Incl Trop Dis. 2019 Jul 29;25:e20190010. doi: 10.1590/1678-9199-JVATITD-2019-0010 (PMC6665320; doi:10.1590/1678-9199-JVATITD-2019-0010)
Supplement: Additional file 1. [file 1678-9199-jvatitd-25-e20190010-s2.pdf]

Supplementary Material to "BthTX-I from *Bothrops jararacussu* induces apoptosis in human breast cancer cell lines and decreases cancer stem cell subpopulation"

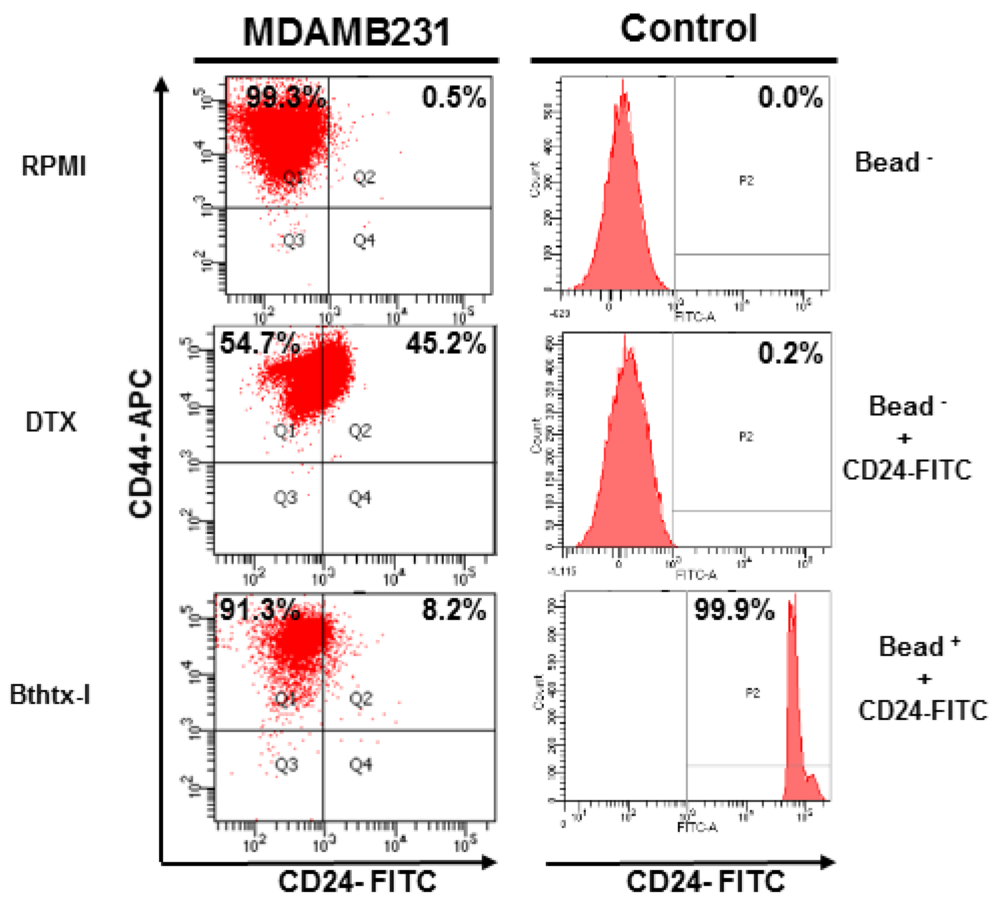

Additional file 2. Flow cytometric analysis of cancer stem cell subpopulation in MDAMB231 cells treated with BthTX-I at 102 µg/mL for 24h. The CD24 and CD44 markers were quantified and antibody testing was performed with Beads (control). DTX: N-desmethyltamoxifen at 20 µM (positive control). RPMI: cells incubated in estrogen-free RPMI 1640 medium supplemented with CS-FBS (negative control).
